# Aquaporin-4 Cell-Surface Expression and Turnover Are Regulated by Dystroglycan, Dynamin, and the Extracellular Matrix in Astrocytes

**DOI:** 10.1371/journal.pone.0165439

**Published:** 2016-10-27

**Authors:** Daniel Kai Long Tham, Bharat Joshi, Hakima Moukhles

**Affiliations:** Department of Cellular and Physiological Sciences, University of British Columbia, Vancouver, British Columbia, Canada; Medical University Vienna, Center for Brain Research, AUSTRIA

## Abstract

The water-permeable channel aquaporin-4 (AQP4) is highly expressed in perivascular astrocytes of the mammalian brain and represents the major conduit for water across the blood-brain barrier. Within these cells, AQP4 is found in great quantities at perivascular endfoot sites but is detected in lesser amounts at the membrane domains within the brain parenchyma. We had previously established that this polarization was regulated by the interaction between dystroglycan (DG), an extracellular matrix receptor that is co-expressed with AQP4, and the laminin that is contained within the perivascular basal lamina. In the present study, we have attempted to describe the mechanisms that underlie this regulation, using primary astrocyte cultures. Via biotinylation, we found that the cell-surface expression of AQP4 is DG-dependent and is potentiated by laminin. We also determined that this laminin-dependent increase occurs not through an upregulation of total AQP4 levels, but rather from a redirection of AQP4 from an intracellular, EEA-1-associated pool to the cell surface. We then demonstrated an association between DG and dynamin and showed that dynamin functioned in conjunction with clathrin to regulate surface AQP4 amounts. Furthermore, we observed that DG preferentially binds to the inactive forms of dynamin, suggesting that this interaction was inhibitory for AQP4 endocytosis. Finally, we showed that laminin selectively upregulates the cell-surface expression of the M23 isoform of AQP4. Our data therefore indicate that the dual interation of DG with laminin and dynamin is involved in the regulation of AQP4 internalization, leading to its asymmetric enrichment at perivascular astrocyte endfeet.

## Introduction

The aquaporins are a family of bidirectional water-permeable channels that are expressed in a wide variety of tissues. AQP4 is the most abundant AQP in the mammalian central nervous system. It is expressed primarily by astroglial cells of the glia limitans externa at the brain surface, the glia limitans interna that line the ventricles, and define the boundaries between the brain and the cerebrospinal fluid, and those at the blood-brain barrier (BBB; [1] [2]). In the BBB, AQP4 is predominantly localized to the perivascular endfeet [3], which are structures that form where astrocytes come into contact with the blood vessels. While animals that lack AQP4 expression exhibit no detectable behavioral defects or gross morphological disruptions in BBB structure [4], they do present deficits in olfaction and audition [5] [6], indicating that the channel may play a central role in brain function. Studies of these animals have also revealed the importance of this channel in cases of severe insults to the brain, such as stroke or injury, in which cytotoxic and vasogenic edema can develop, and often are the main contributors to morbidity or death [7] [8]. The former is caused by the influx of water into the brain through the BBB, which leads to cellular swelling, while the latter is associated with the breakdown of the BBB itself, resulting in the flow of fluid into the extracellular spaces of the brain. AQP4-null mice display improved neurological outcome following water intoxication or ischemic stroke as the accumulation of water in astrocytic endfeet is prevented in the channel's absence, [9], and cytotoxic edema resulting from meningitis is reduced in these animals as well [10]. Paradoxically, vasogenic edema resulting from intraparenchymal fluid infusion, cortical freeze injury, brain tumor and brain abscess was found to be worse in these animals, compared to wild-type controls [11] [12], implicating AQP4 in bulk water clearance. The modulation of AQP4 activity or expression in the brain could therefore result in the amelioration of these disease states.

At the perivascular regions of the brain, AQP4 is coexpressed with DG, α1-syntrophin, dystrophin and dystrobrevin [13] [14] [3] [15] [16], and the evidence linking the function of these components, collectively termed the dystroglycan complex (DGC), to AQP4 expression is quite ample. Indeed, the perivascular localization of AQP4 is lost in the α-syntrophin knockout mouse [13] [17], and the same is to true of dystrophin mutants [18] [19] [20]. The loss of either component also results in a phenocopy of AQP4 knockout, with affected animals displaying a delayed development of cytotoxic brain edema compared to their wild-type littermates [19] [17]. The localization of AQP4 at the perivascular endfeet of glial cells also appears to be crucially dependent on the laminin-binding properties of DG, as seen by the fact that the channel is also mistargeted in Large^myd^ mice, in which the laminin-binding activity of α-DG is ablated due to its defective O-glycosylation [16] [21]. It has been demonstrated that the application of laminin to Müller glia and cortical and hippocampal astrocyte cultures results in the formation of dense clusters of α-DG and AQP4 on the surface of these cells that are analogous to endfoot domains [22] [23]. Further, AQP4 clustering in astrocytes is significantly reduced in instances where DG expression has been disrupted via siRNA. We have additionally observed that laminin-induced clusters of AQP4 and its associated membrane domains exhibit significantly reduced lateral mobility, as measured using the fluorescence recovery after photobleaching assay [24] and a similar reduction of AQP4 mobility was observed in MDCK cells cultured on laminin as well [25]. Based on these data, it has been proposed that the interaction between the DGC and the extracellular matrix (ECM) plays a key role in tethering AQP4 in place at the perivascular astrocyte endfeet. However, the highly asymmetric concentration of AQP4 in astrocyte endfeet may be mediated by mechanisms beyond this alone. Indeed, in the aforementioned study, we established via cell-surface biotinylation that the diffusional retardation caused by laminin in MDCK cells is accompanied by an increased expression of AQP4 at the basolateral plasma membrane [25], implying that laminin-DG interaction could regulate the expression levels of AQP4 at the plasma membrane by altering its internalization. A study published by Zhan *et al*. [26] showing that DG interacts with the GTPase dynamin I in the brain, the latter of which is known to be involved in the scission of newly-formed caveolae and clathrin-coated pits from the plasma membrane, supports this hypothesis. In addition, transferrin receptor internalization, which is mediated by dynamin, is significantly reduced in stem cells null for DG [26].

We therefore chose in the present study to investigate whether DG, in addition to its role as a scaffolding molecule, also serves to extend AQP4’s longevity at the cell surface by suppressing its dynamin-mediated endocytosis in astrocytes. Via a variety of approaches, we determined that the cell-surface expression of AQP4 is dependent on DG, and is potentiated by laminin. We demonstrated that laminin exerts its effect not by increasing the synthesis of AQP4, but rather by suppressing its endocytosis. We observed that DG interacts with caveolin1 and dynamin in astrocytes, and showed that the latter, functioning cooperatively with clathrin, is important in the regulation of AQP4 endocytosis. We also provide evidence indicating that dynamin’s interaction with DG may reduce the availability of the former, and serves to modulate its function. Finally, we saw that laminin selectively upregulates the M23, but not the M1 isoform of AQP4. Based on these lines of evidence, we propose that laminin, DG, and dynamin comprise the primary components of a complex involved in the regulation of clathrin-mediated AQP4 endocytosis, and that their interaction serves to promote the enrichment of M23-based orthogonal arrays of particles at astrocytic endfeet.

## Materials and Methods

### Antibodies

The following antibodies were used: rabbit polyclonal IgG raised against a GST-conjugated polypeptide corresponding to residues 249–323 of rat AQP4 (Alomone Laboratories, Jerusalem, IL), rabbit polyclonal IgG targeting residues 244–323 of human AQP4 (Santa Cruz Biotechnology, California, USA), mouse anti-β-DG, 43DAG1/8D5, specific for 15 of the last 16 amino acids at the C-terminus of the human dystroglycan sequence (Novocastra Laboratories, Newcastle-upon-Tyne, UK), mouse anti-hemagglutinin (HA) IgG (Abgent, California, USA), mouse anti-EEA-1 specific for residues 3–281 of the human protein (BD Biosciences, Ontario, Canada), mouse anti-human TfR (targeting residues 3–28 of the tail region of the protein; Zymed Laboratories Inc./Invitrogen, Ontario, Canada), rabbit anti-caveolin1 made against the N-terminal region of the human protein (Santa Cruz Biotechnology), and mouse anti-dynamin (Hudy-1) recognizing residues 822–838 of human dynamin-1 (EMD Millipore, Ontario, CA).

### Plasmids and Constructs

The M1 and M23-AQP4 chimeras bearing GFP between valines 141 and 142 of the second extracellular loop of the channel protein were generated via overlap extension PCR using a protocol similar to that described by Crane and Verkman [27], and cloned into the expression vector pcDNA3.1 (Invitrogen/Life Technologies, Ontario, Canada).

### Cell Culture

Primary astrocytes used in this study were cultured from the dissected cortices of 1-day-old Sprague-Dawley rat pups (Charles River). In the case of experiments investigating the effects of laminin on AQP4 uptake and distribution, cells were incubated with mouse Engelbreth-Holm-Swarm sarcoma laminin-111 (Sigma-Aldrich, Missouri, USA) at a concentration of 30 nM over a period of approximately 18 hours prior to assays. All transfections were performed using Lipofectamine 2000 (Invitrogen, Ontario, CA) as the means of delivery. Control non-targeting siRNA and siRNA specific for the rat dystroglycan (*Dag1*) gene (ON-TARGETplus SMARTpool; Dharmacon, Illinois, USA) were applied at a final concentration of 100 nM. Assays were performed 48–72 hours following transfection. Viral infections were performed on adherent cells, grown to 80% confluence. As with the transfection experiments, 48–72 hours were allowed to elapse before effects were assessed. Where indicated, cells were treated with 160 μM dynasore monohydrate or 25 μM chlorpromazine hydrochloride (Sigma-Aldrich, Missouri, USA; reconstituted in DMSO and sterile distilled water respectively) for a period of 90 and 30 minutes, respectively prior to assay.

### Cell Surface and Pulse-Chase Biotinylation

In cell-surface biotinylation experiments, primary astrocytes were first washed thrice with ice-cold DPBS, and were then treated with 0.5 mg/ml EZ-Link Sulfo-NHS-LC-Biotin dissolved in DPBS (Pierce Biotechnology, Illinois, USA) for 30 minutes at 4°C. After this, excess biotin was quenched with 50 mM NH_4_Cl in DPBS, then the cells were washed extensively before being incubated for 30 minutes in extraction buffer (25 mM Tris pH 7.4, 25 mM glycine, 150 mM NaCl and 5 mM EDTA) containing 1% Triton X-100 and protease inhibitors (Roche, Quebec, CA). The biotinylated proteins were precipitated using agarose-conjugated streptavidin (Pierce Biotechnology, Illinois, USA). Bound proteins were re-solubilized and denatured using reducing/loading buffer (50mM Tris pH 6.8, 2% SDS, 10% glycerol, and bromophenol blue), and analyzed by SDS-PAGE. For pulse-chase biotinylation, cells were washed in PBS, and then treated with 0.5 mg/ml EZ-Link Sulfo-NHS-SS-Biotin (Pierce Biotechnology, Illinois, USA) in 125 mM NaCl, 2 mM CaCl_2_ and 10 mM TEA for 30 minutes at 4°C, before being washed again and incubated at 37°C with culture medium for 15 and 30 minutes to allow the uptake of biotinylated cell-surface proteins to occur. Biotin bound to proteins not taken into the cell over that time period was cleaved via two successive 15-minute treatments with 50 mM reduced glutathione (Sigma-Aldrich, Missouri, USA) in a buffer consisting of 75 mM NaCl and 75 mM NaOH at 4°C. This was then followed by two more 15-minute incubations with 50 mM iodoacetamide (Bio-Rad, Ontario, CA), dissolved in ice-cold PBS containing 1% BSA. The subsequent cell lysis, protein precipitation, and denaturation steps were carried out using the methods already described.

### Immunofluorescence

Primary astrocyte cultures were washed with DPBS and fixed with 2% PFA. Following additional washes with PBS they were blocked and permeabilized with PBS containing 2% BSA and 0.3% Triton X-100. Labeling was then performed via sequential incubations with primary and fluorophore-conjugated secondary antibodies, diluted 1/100 and 1/700, respectively (Molecular Probes, USA). The cells were then mounted on glass slides using Prolong Gold Antifade reagent (Invitrogen, Ontario, CA), and visualization was performed using an Olympus Fluoview 1000 confocal microscope.

### Quantitative and Statistical Analysis

Densitometric analysis was performed to quantify relative protein amounts across sample sets, and normalizations were performed against the loading controls specified in the text. For the quantitation of colocalization, confocal stacks were collapsed into single images from which the Mander’s coefficients for the overlap of the relevant channels were then calculated via ImageJ (http://rsb.info.nih.gov/ij/). Statistical calculations and Student's *t*-tests were performed using GraphPad Prism 5 (La Jolla, California, USA).

### Ethics Statement

All procedures involving animals in this study were performed in accordance with Canadian Council on Animal Care guidelines, and our protocol was approved by the Animal Care Committee of the University of British Columbia (approval number A06-0319). Upon receipt, dams were separated from their neonates and immediately euthanized using carbon dioxide. Neonates were then decapitated with sharp surgical scissors, and then dissected to obtain the brain tissue used for astrocyte culture. Every effort was undertaken to ensure the humane treatment of research animals, and to minimize their pain and suffering.

## Results

### Laminin Increases Aquaporin-4 Expression at the Plasma Membrane in a Dystroglycan-Dependent Manner

**Fig 1:**
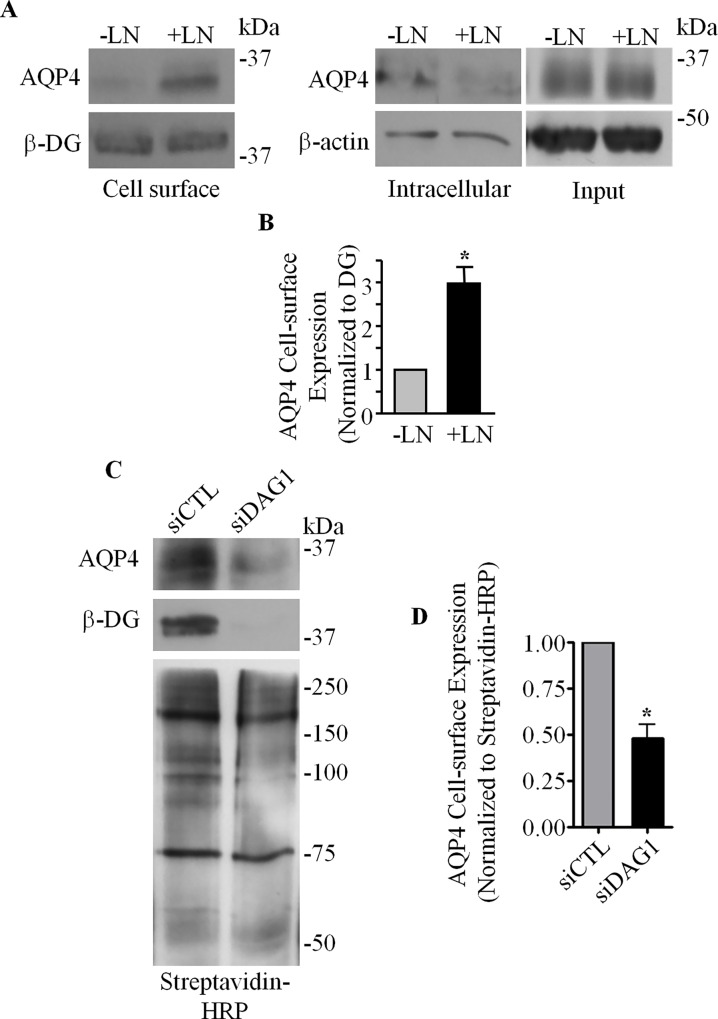
Dystroglycan and laminin are involved in the regulation of AQP4 cell-surface expression in astrocytes. **(A)** The cell-surface, intracellular, and total (Input) fractions from control untreated astrocytes (**-**LN) and astrocytes treated with 24 nM laminin-111 (+LN) were immunoblotted for AQP4 and β-DG. **(B)** Histogram summarizing the differences in protein expression levels of AQP4 at the cell surface of untreated and laminin-treated astrocytes, normalized against β-DG levels. Values represent relative normalized mean pixel intensities ±SEM from three different experiments. The asterisk indicates a statistically significant increase of AQP4 expression compared to untreated astrocytes, as determined by a two-tailed Student’s *t*-test (*p = 0.033). **(C)** Cell-surface proteins isolated via biotinylation assay from siCTL and siDAG1-transfected astrocytes were immunoblotted for AQP4, β-DG, and with streptavidin-HRP to visualize all biotinylated proteins. **(D)** Quantification of cell-surface AQP4 levels, normalized against all biotinylated species, visualized using streptavidin-HRP, for three independent experiments. Asterisk indicates statistical significance, as determined by Students's *t*-test (*p = 0.020).

Our previous finding that exogenous laminin triggers a gain in the expression of AQP4 at the basolateral domain of epithelial MDCK cells when it is present in the culture substrate [25] prompted us to first investigate if laminin would exert a similar effect in astrocytes. Utilizing a biotinylation approach, we determined that primary astrocytes cultured in the presence of exogenous laminin, compared to untreated controls, did indeed exhibit an approximately threefold (2.97 ± 0.37) increase in AQP4 amounts at the plasma membrane (Fig 1A left and B). Consistent with our previous study [25], laminin does not affect the total expression levels of AQP4 (Fig 1A right), but rather affects its localization within the cell. We then asked whether DG plays a role in the cell surface expression of AQP4. Via biotinylation, we found that the siRNA-mediated silencing of DG expression led to a pronounced loss of AQP4 from the cell surface (Fig 1C and 1D). Taken together, these data demonstrate that DG is central in the regulation of AQP4 cell surface localization.

### Laminin Causes the Depletion of AQP4 from Early Endosomes

**Fig 2:**
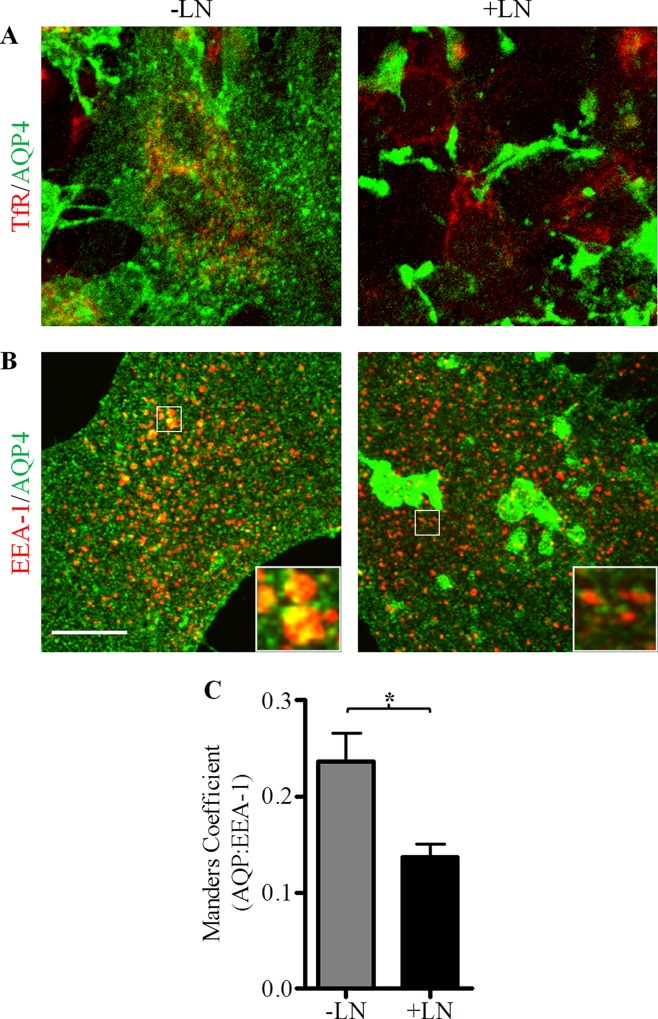
Laminin decreases the amount of AQP4 present in EEA-1-positive intracellular vesicles. Untreated control (-LN) and laminin-treated astrocytes (+LN) were double immunolabeled for AQP4 and transferrin receptor (TfR, **A**) or early endosome antigen 1 (EEA-1, **B**). Images were acquired across 40 successive focal planes via confocal microscopy and then summed to produce Z-stack images (scale bar = 10 μm). **(C)** Histogram summarizing the degree of colocalization ±SEM observed between AQP4 and EEA-1-containing endocytic vesicles quantified from stacks assembled from 40 collapsed images across 9 separate fields of view for -LN and +LN cells. The asterisk indicates a statistically significant decrease of this colocalization in the presence of laminin (+LN), as determined by the two-tailed Student’s *t*-test (*p = 0.0144).

Based on the observation that laminin increases the amounts of AQP4 detected at the cell-surface without causing a concomitant elevation in whole-cell channel levels, we postulated that astrocytic AQP4 is sequestered in intracellular domains in the absence of laminin. To investigate this possibility, we first performed an experiment to determine whether AQP4 colocalizes with markers of intracellular vesicles known to be important in mediating the transport of protein cargoes. Primary astrocytes cultured on glass coverslips were stained with a polyclonal antibody against AQP4, and monoclonal antibodies against the recycling endosomes marker transferrin receptor (TfR) and early endosome antigen-1 (EEA-1; Fig 2).

We saw by confocal microscopy that, among these markers, only EEA-1 possessed any appreciable degree of colocalization with AQP4 (Fig 2B), thereby indicating that a portion of the intracellular pool of AQP4 is found within early endosomes. Laminin appeared to be an important factor in the regulation of this fraction of channels: whereas 21.89 ± 3.05% of AQP4-linked fluorescence was detected in EEA-1-positive vesicles in control cells, this association was virtually halved in cells treated with laminin-111 (Fig 2C), suggesting that the laminin-induced increase in AQP4 cell surface expression may be due in part to the depletion of AQP4 from an endocytic pathway.

### Laminin Inhibits AQP4 Internalization

**Fig 3:**
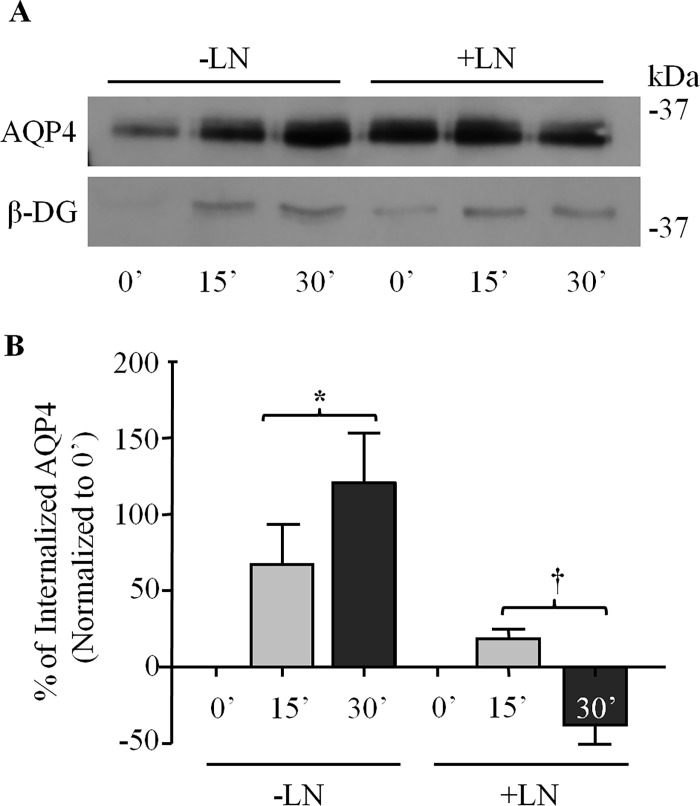
Laminin inhibits AQP4 endocytosis in astrocytes. **(A)** Control untreated (-LN) and laminin-treated astrocytes (+LN) were subjected to pulse-chase biotinylation, and fractions isolated at the time-points indicated were separated via SDS-PAGE and immunoblotted for AQP4 and β-DG. **(B)** Histogram showing the effect of laminin on the rates of AQP4 endocytosis. Values represent the average change, in percent, in the protein levels of internalized AQP4 at each time-point over that seen at 0’ from three different experiments. Symbols indicate statistically significant differences, as determined by two-tailed Student’s *t*-test (*p = 0.017; †p = 0.026).

We next sought to investigate the mechanism underlying the laminin-induced increase of AQP4 cell surface expression. Following our earlier observation that laminin may result in the reapportioning of AQP4 from the intracellular sites to the cell surface, we hypothesized that laminin stabilizes AQP4 at the plasma membrane, by reducing its uptake into the cell. To investigate this hypothesis, we performed pulse-chase biotinylation experiments to assess the effects of laminin on the endocytosis of AQP4. We observed that, in untreated control astrocytes, AQP4 exhibits a high rate of internalization (Fig 3A and 3B). However, when astrocytes are treated with laminin, AQP4 uptake virtually ceases, suggesting that laminin suppresses AQP4 endocytosis. It is noteworthy that, despite our use of two rounds of glutathione/iodoacetamide washes to cleave surface-bound biotin, a residual signal for AQP4, which is exacerbated by the treatment with laminin, can still be seen at the 0-minute time point (Fig 3A). This signal, however, is considerably greater when the glutathione/iodoacetamide steps are omitted, in effect obliterating the expected differences between the 0- and 15-minute (S1 Fig). Through the combined use of these reagents and our normalization process (see experimental procedures), we were able to minimize the contribution of the signal observed at the 0-minute mark.

### Dystroglycan Associates with the Endocytic Components Caveolin1 and Dynamins I and II

**Fig 4:**
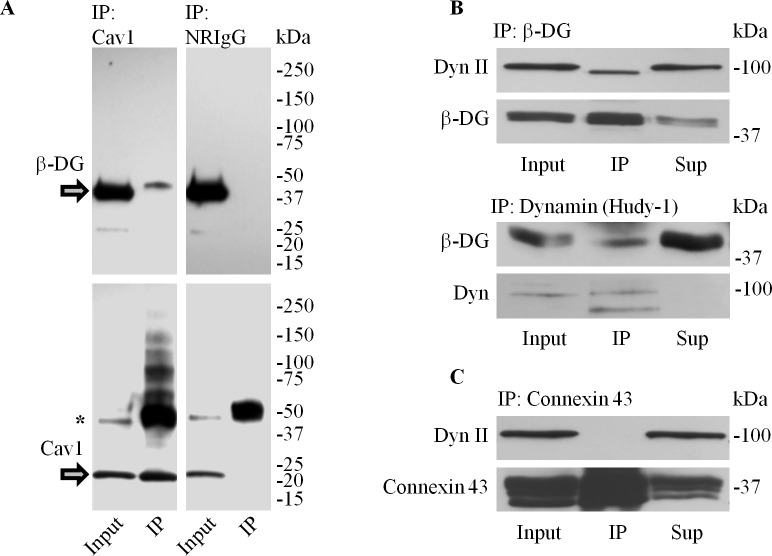
Dystroglycan associates with caveolin1 and dynamin in astrocytes. **(A)** Caveolin1-containing immunoprecipitates were separated via SDS-PAGE and immunoblotted for β-DG and Cav1 (IP). Non-reactive rabbit IgG (NRIgG) was used to control for non-specific binding. Whole cell protein extracts (Input) obtained prior to the addition of protein-G beads were also probed, and are shown alongside for comparison (asterisk in bottom panel indicate cross-reativity of the secondary antibody with the IgG heavy chains). **(B)** β-DG (top panel) and dynamin-containing immunoprecipitates (bottom panel) were immunoblotted for dynamin II and β-DG. **(C)** Connexin 43 immunoprecipitates immunoblotted for dynamin were used as a negative control against the possible non-specific interaction between the intracellular domains of transmembrane proteins and dynamin. Input and unprecipitated proteins (Sup) were also immunoblotted for the indicated proteins.

The effect of laminin on AQP4 endocytosis, together with the fact that DG depletion leads to the loss of channel expression at the cell surface, led us to next ask whether and how DG might be involved in the regulation of AQP4 turnover. As previous studies have demonstrated that the endocytic components caveolin3 [28] and dynamin [26] interact with DG in smooth muscle and brain respectively, we therefore chose to investigate if equivalent links existed in astrocytes. We showed that caveolin1 immunoprecipitates contain β-DG (Fig 4A). This, however, was not seen when non-reactive serum was used instead (Fig 4A). Similarly, dynamin II and β-DG co-immunoprecipitate, whereas connexin-43 immunoprecipitates do not contain dynamin II (Fig 4B), showing that the association between dynamin II and β-DG is specific.

### Dynamin and Clathrin Regulate AQP4 Turnover

**Fig 5:**
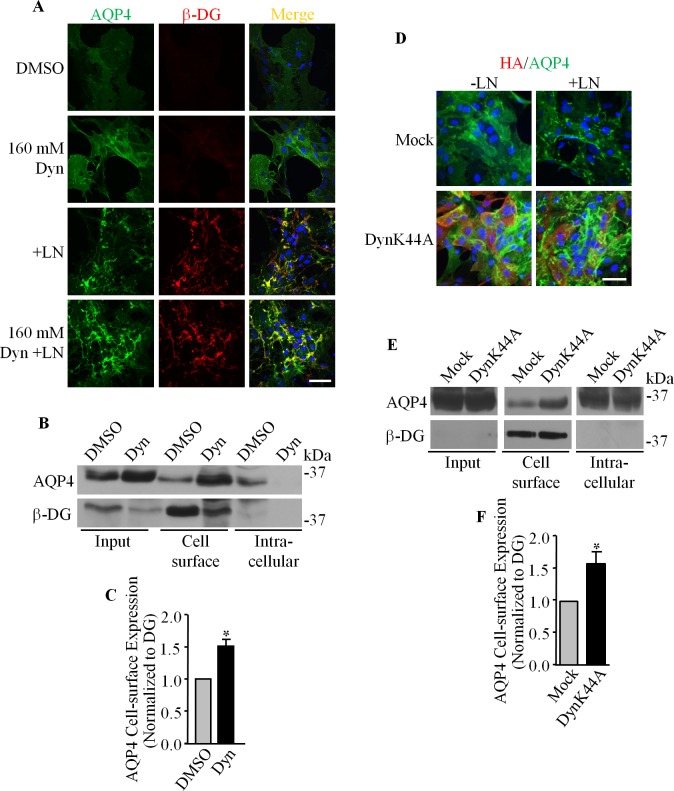
The inhibition of dynamin leads to the accumulation of AQP4 at the cell surface. **(A)** Primary astrocyte cultures were either treated with DMSO, treated with dynasore monohydrate (Dyn), laminin (+LN), or both, before being double immunolabeled for AQP4 and β-DG. **(B)** Cell-surface biotinylation was performed to assess the impact of dynamin inhibition on AQP4 and β-DG expression at the plasma membrane. Whole cell lysates (Input) and unbound proteins (Intracellular) were also immunoblotted for these proteins. A representative immunoblot of AQP4 and β-DG cell surface expression in dynasore and DMSO-treated astrocytes. **(C)** The histogram represents the mean pixel intensity ±SEM of AQP4 cell surface expression normalized to that of β-DG from three different experiments. The asterisk indicates a statistically significant difference compared to DMSO-treated astrocytes, as determined by a two-tailed Student’s *t*-test (* p = 0.0456). **(D, E and F)** Equivalent experiments were carried out on astrocyte cultures infected with adenoviruses carrying a construct for HA-tagged dominant-negative dynamin (DynK44A) and mock-infected astrocytes (Mock). The asterisk in F indicates a statistically significant difference from mock-infected astrocytes, as determined by two-tailed Student’s *t*-test (*p = 0.0472). Scale bars = 50 μm.

**Fig 6:**
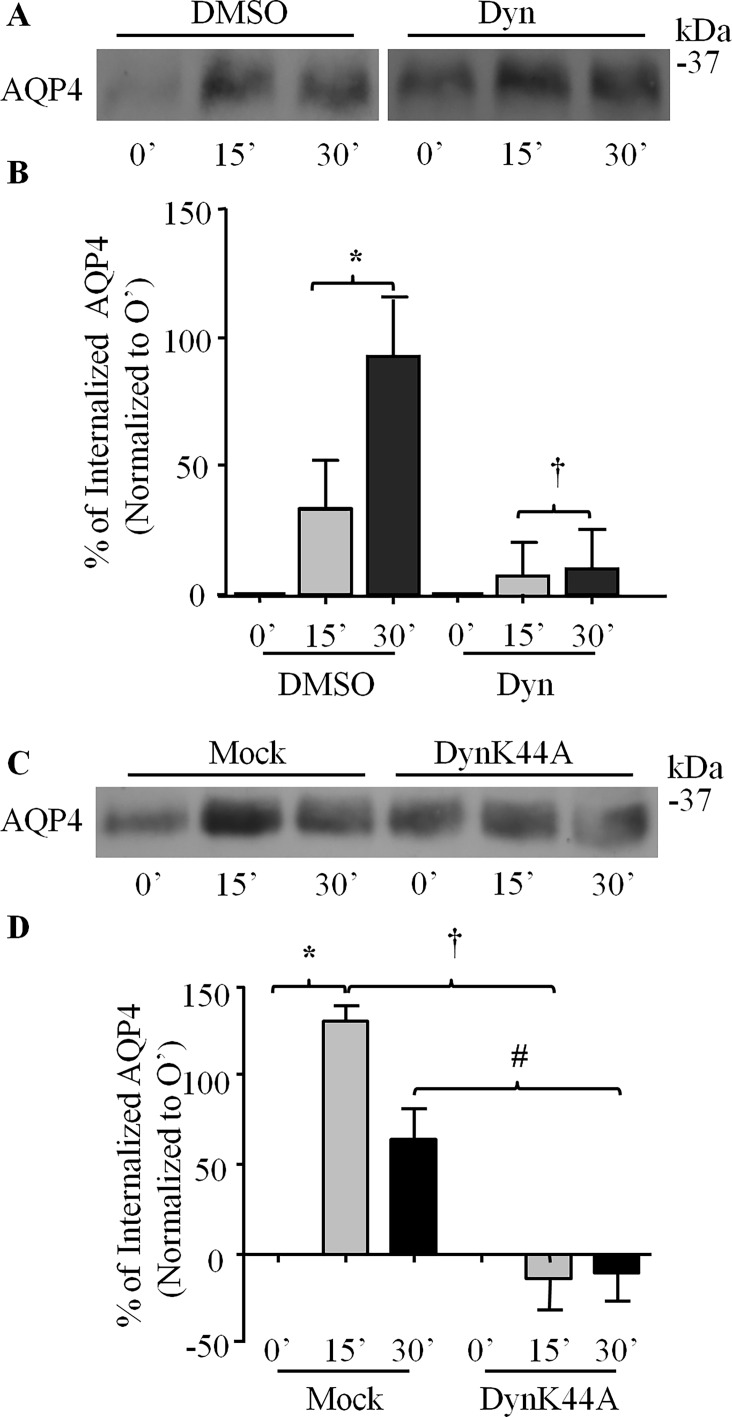
The endocytosis of AQP4 is regulated by dynamin. **(A)** DMSO- and dynasore-treated astrocytes (Dyn) were subjected to pulse-chase biotinylation, and fractions isolated at the time-points indicated were separated via SDS-PAGE and immunoblotted for AQP4. **(B)** Histogram showing the effect of dynasore on the rates of AQP4 endocytosis. Values represent the average percent increases in the levels of internalized AQP4 at each time-point over that seen at 0’ from five separate experiments. Symbols indicate statistically significant differences, as determined by two-tailed Student’s *t*-test (*p = 0.0150; †p = 0.0470). **(C and D)** Equivalent experiments were performed on astrocytes infected with adenoviruses carrying a construct expressing dominant-negative dynamin (DynK44A) and mock-infected astrocytes (Mock). Symbols in D, which summarizes the results of five such experiments, indicate statistically significant differences, as determined by two-tailed Student’s *t*-tests (*p = 0.0214; †p = 0.0006; #p = 0.0003).

Given dynamin’s well-established role in mediating endocytic processes, its association with DG, and the role of DG in the clustering of AQP4 at the plasma membrane of astrocytes, we next chose to investigate the involvement of dynamin in the DG/laminin-mediated increase of AQP4 cell expression. Astrocytes cultured on glass coverslips were treated with 160 μM dynasore monohydrate for 90 minutes and processed for immunofluorescence and confocal imaging. We found that the fluorescence intensity of AQP4 was increased in dynasore-treated astrocytes (Fig 5A), and that laminin induces the formation of more AQP4 clusters in dynasore-treated astrocytes compared to astrocytes treated with laminin alone (Fig 5A). Cell surface biotinylation shows a significant increase of AQP4 cell surface expression in dynasore-treated cells (Fig 5B), corroborating our immunofluorescence results (160 μM Dyn; Fig 5A). Taken together, these findings demonstrate that the GTPase activity of dynamin, which is central to its ability to sever nascent endocytotic vesicles from the plasma membrane, is indeed crucial for the regulation of surface AQP4 levels. Using a pulse-chase approach, we then observed that the treatment of astrocytes with dynasore monohydrate decreases the amount of AQP4 uptake to 9.63 ± 15.76% at 30 minutes, as opposed to 92.55 ± 22.61% for control cells (Fig 6A and 6B).

To more directly test for dynamin’s involvement, we infected astrocytes with an adenovirus carrying a HA-tagged dominant-negative K44A mutant of dynamin II. As in the case of astrocytes treated with dynasore, we found that this GTPase-deficient dynamin halts AQP4 uptake, as reflected by the greater amounts of AQP4 at the cell surface (Fig 5D, 5E and 5F). By pulse chase biotinylation, we were able to demonstrate that this increased expression most probably stemmed from the inhibition of AQP4 internalization, which fell from an average of 130.25 ± 8.32% after 15 minutes for control cells to near-zero levels in dynamin K44A-expressing cells at the equivalent time point (-11.88 ± 16.47%; Fig 6C and 6D). Taken together, these data strongly indicate that the regulation of surface AQP4 levels in astrocytes is dependent on the GTPase activity of dynamin.

**Fig 7:**
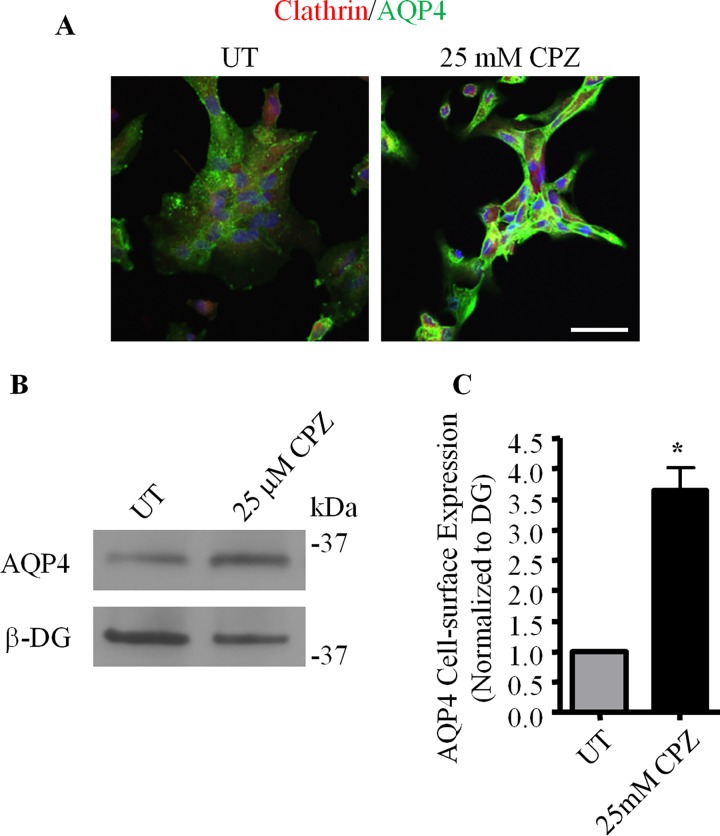
Clathrin regulates AQP4 expression at the cell surface. Untreated control astrocytes (UT) and astrocytes treated with 25 μM chlorpromazine (25 μM CPZ) were **(A)** double-immunolabeled for AQP4 and clathrin heavy chain, and imaged using confocal microscopy or **(B)** subjected to cell-surface biotinylation followed by SDS-PAGE, and immunoblotted for AQP4 and β-DG. **(C)** Histogram illustrating the changes seen in the cell-surface expression of AQP4, as normalized against that of β-DG, over four different experiments. The asterisk indicates a statistically significant difference, as determined by the Student’s *t*-test (*p = 0.0069).

We next investigated if clathrin was also involved in AQP4 endocytosis. To do this, astrocytes were treated with 25 μM chlorpromazine hydrochloride, a pharmacological inhibitor of clathrin-mediated endocytosis [29], for a period of 30 minutes, and then labeled for AQP4 and clathrin. Compared to untreated controls, astrocytes treated with chlorpromazine express higher levels of AQP4 at the cell surface, as seen in the sharp increase in fluorescence at the peripheral regions of these cells (Fig 7A). Additionally, cell-surface AQP4 levels, as assessed by biotinylation (Fig 7B and 7C), were seen to increase 2.92 ± 0.75 times in the presence of chlorpromazine. AQP4 endocytosis thus appears to occur through a dynamin- and clathrin-dependent pathway.

**Fig 8:**
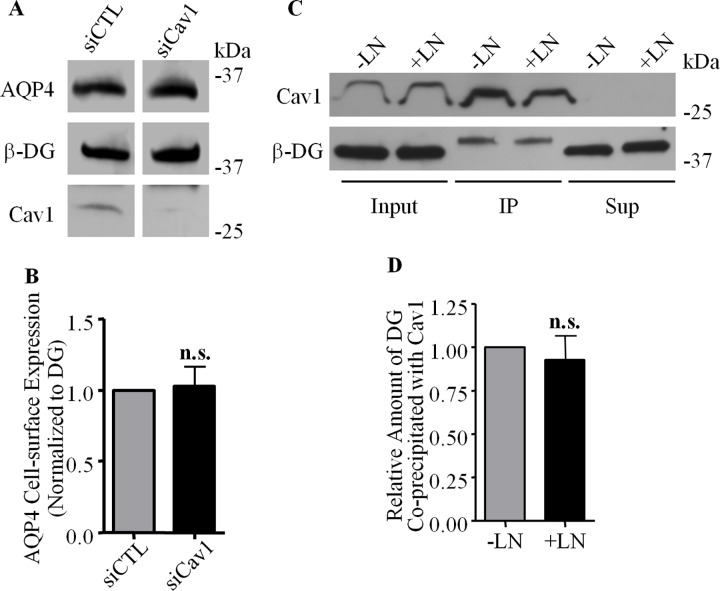
Caveolin1 is not involved in AQP4 turnover. **(A)** Cell surface proteins were isolated via biotinylation from astrocytes treated with siRNA targeting caveolin1 (siCav) and control siRNA (siCTL), and analysed via SDS-PAGE. **(B)** Histogram showing the relative amounts of AQP4 expressed at the plasma membrane in both siCTL and siCav, normalized against β-DG (n = 4; n.s. = not significant). **(C)** Caveolin1-containing immunoprecipitates were isolated from control (-LN) and laminin-treated (+LN) astrocytes, analysed via SDS-PAGE, and the input, immunoprecipitate (IP) and supernatant (Sup) fractions were probed for caveolin1 and β-DG. **(D)** Histogram comparing the amounts of β-DG co-immunoprecipitated with caveolin1 (n = 3; n.s. = not significant).

Caveolin1, however, does not play a role in AQP4 uptake, despite having been identified earlier as an interacting partner of DG. Silencing caveolin1 expression in cultured astrocytes via the transfection of a specific siRNA construct did not alter the cell-surface expression of AQP4 or β-DG compared to control cells transfected with a control scrambled siRNA (Fig 8A and 8B). Furthermore, the interaction between caveolin1 and β-DG does not appear to be regulated by laminin, as evidenced by our observation that identical amounts of β-DG are co-immunoprecipitated with caveolin1 whether or not cells are cultured in the presence of laminin (Fig 8C and 8D).

### Dystroglycan Preferentially Associates with GTP-Depleted Forms of Dynamin

**Fig 9:**
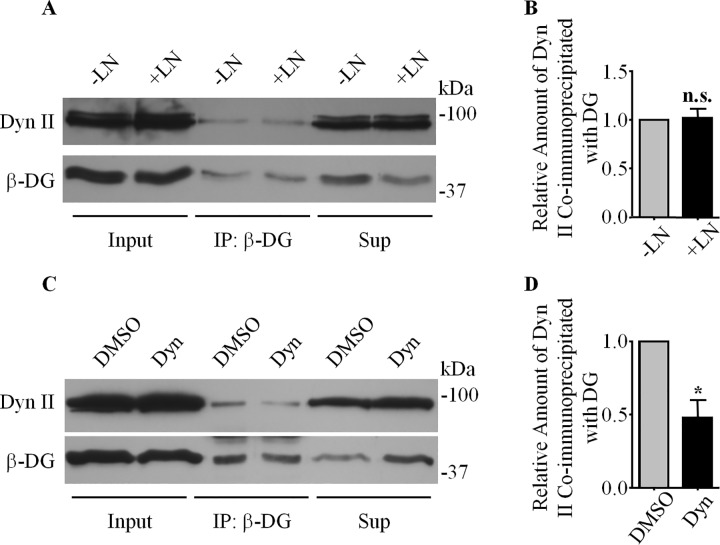
The DG-Dynamin II interaction is modulated by dynamin's activity, but not by laminin binding. **(A)** β-DG-containing immunoprecipitates prepared from astrocytes treated with 160 μM dynasore monohydrate (Dyn), an inhibitor of the GTPase activity of dynamin, or DMSO (used here as a control) were analyzed by SDS-PAGE and then immunoblotted for the dynamin II and β-DG. Whole cell protein extracts (Input) and fractions of proteins left in the supernatant after the immunoprecipitation (Sup) were also immunoblotted for dynamin II and β-DG. **(B)** Histogram representing the mean pixel intensities ±SEM of the dynamin II signal normalized to that of β-DG from five different experiments, with the asterisk indicating a statistically significant difference compared to DMSO-treated astrocytes, as determined by a two-tailed Student’s *t*-test (*p = 0.0119). **(C)** Similar immunoprecipitation experiments were performed on laminin-treated and untreated control astrocytes. **(D)** Quantitation of the relative amounts of dynamin II co-immunoprecipitated with β-DG from cells subjected to either treatment (n.s. = not significant).

While we found that DG interacts with components of the endocytic pathway, the exact nature of this interaction was as yet unclear. Thus, we performed a series of experiments designed to investigate factors important in regulating this interaction. Given laminin’s role in inhibiting AQP4 endocytosis, we tested whether it might regulate the degree of association between dynamin II and β-DG, by probing β-DG immunoprecipitates obtained from astrocytes treated with laminin and untreated controls for dynamin II. We found no significant changes in the amount of dynamin II (Fig 9A and 9B), suggesting that laminin’s effects on AQP4 turnover and localization are not mediated directly by the modulation of DG-dynamin binding. However, the affinity of dynamin for DG appears to be dependent on the state of dynamin. As assessed by immunoprecipitation, astrocytes treated with dynasore monohydrate, a specific inhibitor of the GTPase activity of dynamin [30], exhibited a lower degree of binding between dynamin and DG compared to control cells treated with DMSO only (Fig 9C and 9D). As the treatment with the drug would lead to the accumulation of GTP-bound dynamin, this result suggests that DG preferentially binds to the GDP-bound or unoccupied forms of dynamin, and that this interaction may inhibit the function of dynamin by sequestering it in this inactive form.

### Laminin Regulates the Cell-Surface Expression of the M23 Isoform of AQP4, but Not That of the M1 Isoform

**Fig 10:**
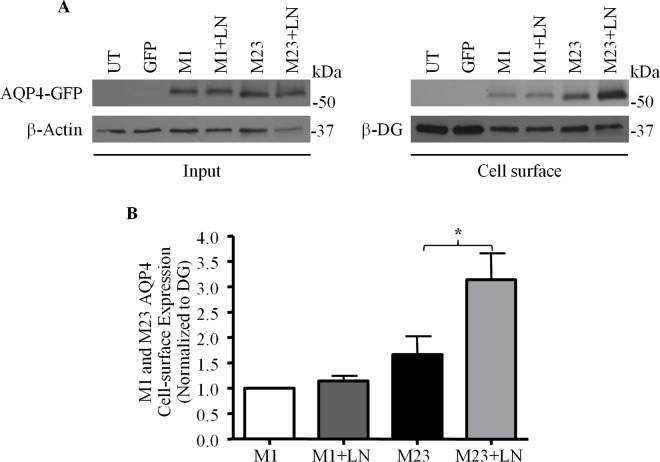
Laminin affects the cell-surface expression of only the M23 isoform of AQP4. **(A)** Laminin-treated (+LN) and untreated control CHO cells expressing GFP-tagged M1 and M23 isoforms of AQP4 were subjected to biotinylation analysis, and the input and biotinylated (Cell surface) fractions were isolated and probed for the proteins indicated. **(B)** Histogram summarizes the results obtained over 4 such experiments. The asterisk indicates a statistically-significant difference, as determined via the Students *t*-test (*p = 0.0408).

Two major isoforms of AQP4 are expressed in astrocytes: M1, which is approximately 34 kDa, and the smaller 31 kDa M23 isoform, which differs from the former by a 22 amino acid truncation in its N-terminus [31]. Whereas the M1 isoform exists primarily as singular tetramers that infrequently assemble into groups of no more than 12 similar subunits, tetramers of M23 organize into massive orthogonal arrays of particles (OAPs) comprised of more than one hundred linked subunits [32]. The relative abundance of M1 and M23 is thus a critical determinant of OAP dimension. Because of the potential functional implications of this, particularly with respect to channel distribution and organization at the astrocytic-vascular interface, we decided in our next experiment to investigate if the ECM may be a factor that governs how each of these isoforms is expressed. To do so, we expressed M1 or M23 AQP4 bearing GFP tags in each of their second extracellular loops in two sets of CHO cells, which were then treated with laminin. Via biotinylation, we then compared the cell-surface amounts of AQP4 in these cells with that of untreated controls also expressing these tagged AQP4 constructs. We found that, in the absence of laminin, M23-AQP4 is expressed at higher levels at the cell surface compared to the M1 isoform, despite the fact that M1 and M23 AQP4 are detected in equivalent amounts in the input fraction, thereby indicating that M23 possesses a greater tendency to be localized at the plasma membrane (Fig 10A), which may in turn explain the observed predominance of that isoform at the surface of astrocytes. Further, while M1 poses a near-negligible response to exogenous laminin, cells expressing M23-AQP4 exhibit a doubling of the amounts of this isoform at the plasma membrane (Fig 10A and 10B). These data show that laminin upregulates cell surface expression of M23-AQP4 but not that of M1-AQP4.

## Discussion

In the mammalian brain, AQP4 is highly-concentrated at the perivascular endfoot domains of astrocytes, where these cells come into contact with the laminin-rich perivascular basal lamina [15]. While the reasons for this are not completely understood, our previous finding that treating astrocyte cultures with exogenous laminin causes dense clusters comprised of AQP4, laminin, and DG to form [22] suggested that the DG complex might tether AQP4 in place at the endfoot via its affinity for laminin. We show in the present study that exogenous laminin, acting via DG, causes AQP4 cell-surface expression to be upregulated also, indicating that DG is important not just for the localization of AQP4 at perivascular endfoot domain, but the enrichment of this channel at these sites as well.

As laminin treatment does not affect global AQP4 amounts, it appears that this enrichment arises not through an increase in the *de novo* synthesis of channels, but rather from a reapportioning of existing channels within the cell. This hypothesis is further supported by our observation that the increase in plasmalemmal AQP4 levels is concomitant with the depletion of the channel from EEA-1-positive early endosomes. This prompted us to further investigate the question of whether laminin might impinge on AQP4 endocytosis. Via pulse-chase biotinylation, we saw that while surface AQP4 is rapidly internalized in untreated control cells, laminin severely suppresses channel internalization. The strikingly divergent fates of AQP4 seen here may indicate that the polarized distribution of AQP4 *in vivo* may be the combined result of opposing forces that act to retain the channel at the endfoot, while clearing it from other domains.

Via immunoprecipitation assays, we determined that DG interacts with both caveolin1 and dynamin, the latter of which we then demonstrated to be directly involved in the regulation of AQP4 endocytosis, and, consequently, its cell-surface expression. We subsequently found that the inhibition of the GTPase activity of dynamin in astrocytes via the use of the pharmacological compound dynasore monohydrate effectively halved the amount of dynamin co-immunoprecipitated with DG, which in turn pointed to the fact that DG might preferentially associate with the “inactive” GDP-bound form of dynamin, or its guanosine-depleted form. The above result raised the possibility that the laminin-DG interaction may regulate the sequestration of dynamin, and through this, moderate the rate of endocytosis. These results are in agreement with, and expand upon, that of Zhan *et al*. [26], who showed that the knock-in of DG into DG-null embryonic stem cells dramatically reduces transferrin receptor internalization. We also found that chlorpromazine hydrochloride, a cholestatic compound that inhibits clathrin-mediated endocytosis [29], similarly increases AQP4 cell-surface expression, consistent with the well-characterized cooperative roles of dynamin and clathrin [33], and the fact that AQP4 endocytosis involves the clathrin adaptor protein 2 complex [34]. These results indicate that the rapid turnover of AQP4 in control cells not treated with laminin is mediated by a clathrin- and dynamin-dependent endocytic mechanism. More interestingly, they also imply that DG is the conduit through which laminin exerts its inhibitory effects on AQP4 internalization, serving to selectively downregulate dynamin function where the former is present.

As mentioned, exogenous laminin causes clusters of AQP4 to form [22]. We also observed in this study that laminin preferentially increases the cell-surface expression of the M23 isoform of AQP4 over that of the M1 isoform, raising the possibility that clusters could be composed of OAPs. Given their considerable dimensions, it is likely that they might be less amenable to endocytic uptake, a possibility that is in agreement with their relatively stable diffusional dynamics [24], and further underscored by our finding in the present study that laminin treatment depletes a significant fraction of AQP4 from early endosomes. How then, does DG translate the binding of laminin into increased AQP4-M23 levels at the plasma membrane of cultured astrocytes? Our finding that laminin has no effect on the degree of association between DG and dynamin or the overall cell-surface amounts of DG suggest that laminin probably does not potentiate the inhibition of dynamin function. That is to say, the increase in AQP4 seen cannot be the product of a global deceleration in endocytic rates. Laminin does, however, cause DG to cluster, which could create zones in which dynamin-mediated endocytosis is significantly suppressed. Within such a zone, newly-exocytosed M23 subunits would be protected from immediate re-uptake, thereby permitting their eventual accretion into OAPs, and then into clusters. The role of laminin therefore, in this instance, is to act as the spatial cue that directs where OAPs coalesce.

**Fig 11:**
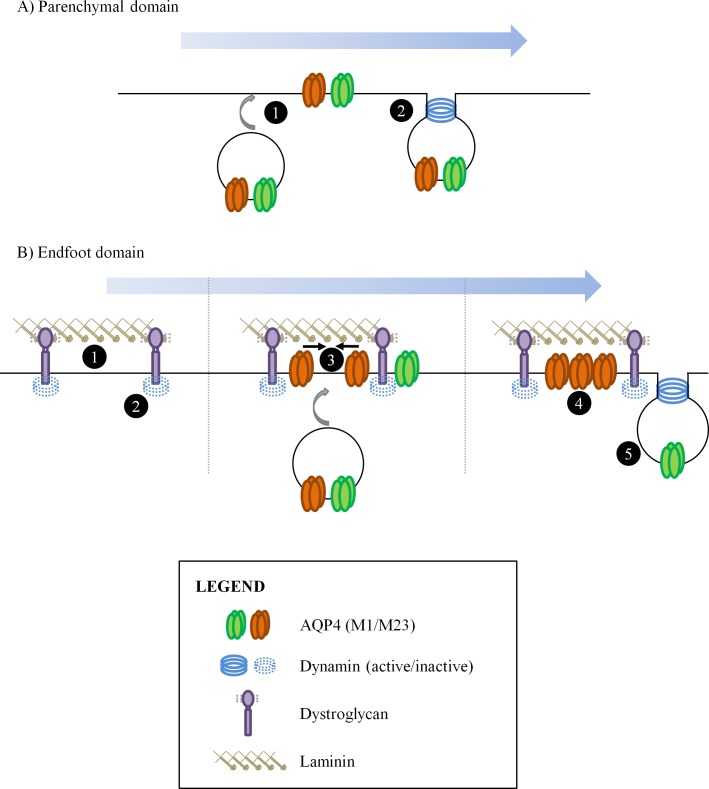
Regulation of cell surface AQP4 levels at the parenchymal and endfoot domains of astrocytes. **(A)** AQP4 channels appearing at the cell surface (1) at parenchymal domains are rapidly taken in via a clathrin- and dynamin-dependent mechanism (2). The accumulation of large amounts of this channel is thereby prevented. **(B)** At the endfoot regions however, the presence of laminin in the basal lamina causes DG to cluster tightly (1), suppressing dynamin activity in the immediate vicinity (2). AQP4 channels being transported to the plasma membrane are thus protected from being endocytosed, which allows M23-based channels to begin to accrete into OAPs (3). Being large, these OAPs are resistant to dynamin-mediated endocytosis, which continues to operate on AQP4-M1-based channels.

It is likely this is laminin's function at the perivascular endfoot as well. We hypothesize that the asymmetry in AQP4 distribution seen in perivascular astrocytes is generated thusly (Fig 11): a) within the parenchymal domains of astrocytes, AQP4 channels, regardless of type, are continually turned over as they are brought to the cell surface but rapidly taken back via clathrin-coated pits, facilitated by dynamin. In this manner, levels of the channel are kept relatively low (Fig 11A), and b) At the endfoot however, DG recruited into clusters via its affinity for the laminin present in the perivascular basal lamina binds to and maintains dynamin in an inactive form, thereby creating an area of decreased clathrin-mediated endocytosis. As such, AQP4-M23-based channels just appearing at the surface are afforded the opportunity to accrete and form large, stable OAPs that are resistant to uptake because of their dimensions, and their association with the DG complex. These accumulate over time, resulting in the endfoot being enriched for AQP4. Channels comprised of AQP4-M1, which are excluded from large OAPs, are not protected (Fig 11B).

Previous studies on the involvement of the DGC in mediating AQP4 distribution in astrocytes have primarily cast their focus on its function in the tethering of these channels to astrocytic endfeet [15] [22] [3] [16]. In the present study, we have provided evidence to demonstrate that DG, via its interactions with both the perivascular ECM and dynamin, also serves to facilitate the enrichment of the channel within these cellular domains.

## Supporting Information

S1 Fig Glutathione significantly reduces the detection of cell-surface AQP4 channels. When the reducing agent glutathione is used to break the disulfide bond in the cleavable biotin analog used in the pulse-chase protocol, the contribution of cell-surface AQP4 to the total signal is sharply reduced, and a clear difference can be seen between the 0-minute and 15-minute samples (left panel top). When this step is ommitted, however, the signal originating from the cell-surface pool becomes so high as to render the differences between the two time-points undetectable (right panel top). The fact that the signal for dystroglycan, which appears to experience a relatively low degree of turnover relative to AQP4 (left bottom), becomes as intense as that for AQP4 in the absence of glutathione is further indication of the effectiveness of this cleavage step.
(TIF)
