# Supplementary figures and images for: Aquaporin-4 Cell-Surface Expression and Turnover Are Regulated by Dystroglycan, Dynamin, and the Extracellular Matrix in Astrocytes
Source: PLoS One. 2016 Oct 27;11(10):e0165439. doi: 10.1371/journal.pone.0165439 (PMC5082936; doi:10.1371/journal.pone.0165439)

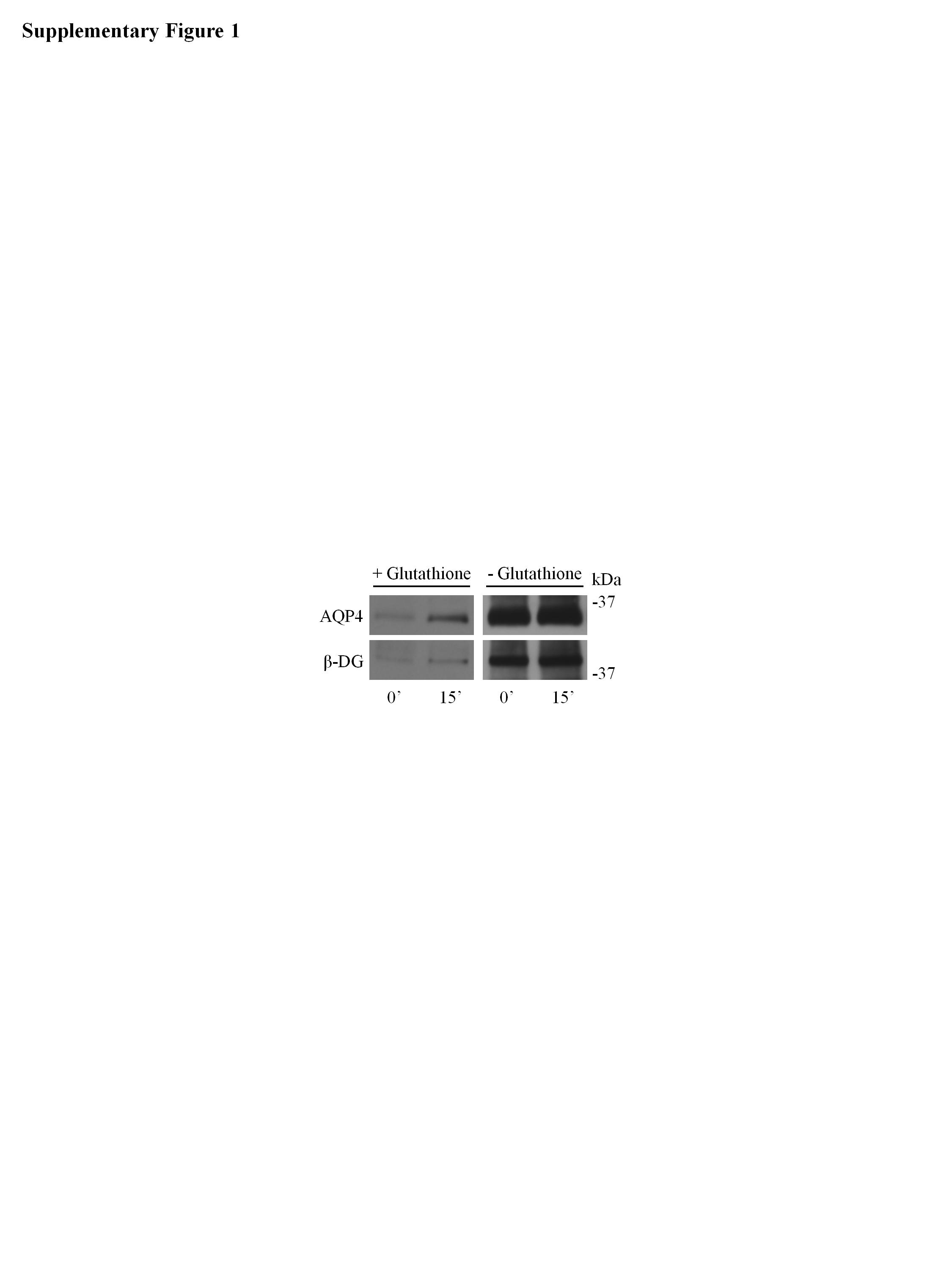

Supplement: S1 Fig — When the reducing agent glutathione is used to break the disulfide bond in the cleavable biotin analog used in the pulse-chase protocol, the contribution of cell-surface AQP4 to the total signal is sharply reduced, and a clear difference can be seen between the 0-minute and 15-minute samples (left panel top). When this step is ommitted, however, the signal originating from the cell-surface pool becomes so high as to render the differences between the two time-points undetectable (right panel top). The fact that the signal for dystroglycan, which appears to experience a relatively low degree of turnover relative to AQP4 (left bottom), becomes as intense as that for AQP4 in the absence of glutathione is further indication of the effectiveness of this cleavage step. (TIF) [file pone.0165439.s001.TIF]
